# Mid-term impact of patient-prosthesis mismatch on young and middle-aged patients undergoing surgery due to severe aortic stenosis

**DOI:** 10.1186/1749-8090-8-S1-O101

**Published:** 2013-09-11

**Authors:** D Hernandez-Vaquero, R Diaz, JM Garcia, D Calvo, JC Llosa

**Affiliations:** 1 Cardiac Surgery Department, Central University Hospital of Asturias, Oviedo, Spain; 2 Department of Cardiology, Central University Hospital of Asturias, Oviedo, Spain

## Background

The clinical impact of patient-prosthesis mismatch on outcomes in young and middle-aged patients undergoing surgery for aortic valve replacement remains unknown. Our objective was to examine the mid-term impact of patient-prosthesis mismatch on overall mortality, quality of life and cardiac events.

## Methods

All patients younger than 70 years of age undergoing isolated aortic valve replacement from October 2005 to October 2011 were analyzed. Patient-prosthesis mismatch was defined as the indexed effective orifice area ≤ 0,85cm2/m2. We studied the impact of patient-prosthesis mismatch on mid-term overall mortality, cardiac events and New York Heart Association functional class using an analysis stratified for propensity score. Cardiac events were defined as cardiac death, sudden death, hospital readmission due to angina, syncope or heart failure or reoperation on aortic prosthesis.

## Results

293 patients were included in the study, of whom 81 (27,61%) had some degree of patient-prosthesis mismatch. Patient-prosthesis mismatch had no impact on mid-term overall mortality (HR=1,45 CI 95%= 0,65-3,22; p=0,36), although it had a negative impact on cardiac events (HR=11,52 CI 95%=5,25-25,24; p<0,001) and functional class (RR=7,55 CI 95%=2,59-22,03; p<0,001).

## Conclusions

Patient-prosthesis mismatch appears to be a strong and independent predictor of cardiac events and functional class in young and middle aged patients undergoing aortic valve replacement for severe stenosis. However, it is possible that it has no impact on overall mortality.

